# Terahertz bistatic three-dimensional computational imaging of hidden objects through random media

**DOI:** 10.1038/s41598-024-56535-y

**Published:** 2024-03-14

**Authors:** Quanchun Yu, He Cai, Xianli Zhu, Zihao Liu, Hongcheng Yin, Liangsheng Li

**Affiliations:** National Key Laboratory of Scattering and Radiation, Beijing, 100854 People’s Republic of China

**Keywords:** Terahertz optics, Imaging and sensing

## Abstract

Random media pose limitations on the imaging capability of photoelectric detection devices. Currently, imaging techniques employed through random media primarily operate within the laser wavelength range, leaving the imaging potential of terahertz waves unexplored. In this study, we present an approach for terahertz bistatic three-dimensional imaging (TBTCI) of hidden objects through random media. By deducing the field distribution of bistatic terahertz time-domain spectroscopy system, and proposing an explicit point spread function of the random media, we conducted three-dimensional imaging of hidden objects obscured by the random media. Our proposed method exhibits promising applications in imaging scenarios with millimeter-wave radar, including non-invasive testing and biological imaging.

## Introduction

The limitations brought by the existence of random media are widely observed from industrial inspections to medical diagnosis^1–4^. For example, existing optoelectronic imaging devices face difficulties in accurately recovering the original appearance in the application such as material defect detection and security inspection^5–7^. Underwater imaging remains a challenge in hydrographic exploration^8–10^, while imaging through surface biological tissues poses difficulties for medical professionals^11–16^.

Scattering phenomena occur across various spectrums. With the transmission environment primarily being the atmosphere, random media in this context mainly comprise small water droplets and aerosol particles, typically on the scale of micrometers or smaller, this size range approximates the wavelength of visible light and infrared waves. As a result, scattering phenomena are pervasive in the optics band, leading to lots of research on imaging through random media. To our knowledge, there are four main approaches to address this issue. The first method involves selectively imaging using ballistic photons^17–19^. When light propagates through random media, there is a chance that some photons will not be scattered by the media. These photons are referred to ballistic photons. Ballistic photons propagate almost in a straight line and arrive ahead of other photons that pass through random media. To accurately capture ballistic photons, one approach is to employ time gating techniques^18,19^. Time gating technique sets an appropriate temporal gate to exclusively capture the desired light signals and reject those outside the designated time range. Another effective approach is coherent gating technique^17,20,21^. Coherent gating is a technique employed in optical coherence measurements to measure the intensity distribution of light returning from different depths by controlling the optical path length of the reference light. These methods bypass the effects of scattering but results in significant loss of photon utilization, especially when detecting ballistic photons in thick scattering media or media with strong scattering properties, it will be a severe challenge of detector sensitivity. The second approach involves calculating higher-order correlations of the scattering field^22–26^, which does not require dealing directly with the troublesome random media. The effectiveness of calculating higher-order correlations depends on the range of memory effects, and often have strict requirements, such as the requirement for the random media to remain static or the restriction on the movement range of the light source. However, recent advancements in research have overcome these constraints, rendering correlation imaging methods more stable and versatile^25,26^. The third approach establish the connection between image degradation before and after by designing complex and massive neural networks. This method typically requires a large amount of annotated data for training, which in turn demands significant computational resources and time. However, from traditional convolutional neural networks to recent generative adversarial networks, deep learning methods have demonstrated increasing proficiency in the field of imaging through scattering media area^27–33^. Finally, the method of accurately simulating the point spread function of random media has the advantages of reliability and low computational complexity, and thus holds significant potential for applications in the field of imaging through random media^34,35^. This method relies on extensive prior knowledge as support, usually requiring preliminary research to determine the mathematical form of the point spread function and parameter values through pre-calibration. In the terahertz wave and longer wavelength wave (wavelengths greater than 1 mm), water droplets and aerosol particles in the atmosphere have minimal impact. Consequently, imaging through random media is rarely discussed in terahertz wave bands. However, in various fields such as industrial inspection and medicine, synthetic fibers, plastic materials, and biological tissues still exhibit strong scattering effects on terahertz waves. This poses a significant challenge for terahertz detection systems in terms of computational imaging algorithms.

In addition, it is common for imaging through random media algorithms to be designed based on monostatic detection systems. However, to prevent signal aliasing, the monostatic terahertz radar usually uses a transceiver to control the emission and reception of terahertz pulses or a beam splitter to change the direction of terahertz wave propagation. These lead to an increase in measurement time or a decrease in received signal intensity.

In this study, in order to enhance the imaging capabilities of terahertz waves through random media and overcome the efficiency limitations associated with monostatic radar, we introduce a method for terahertz bistatic three-dimensional imaging (TBTCI) of hidden objects through random media. We model and invert the scattering of terahertz waves that travel through random media, propagate through free space to a hidden object, scatter back, and travel through random media again. We designed multiple random media occlusion scenarios and tested our method with various hidden object types, and in all cases, the hidden object images were recovered. 

## Experiment and methods

### Experimental setup

**Figure 1:**
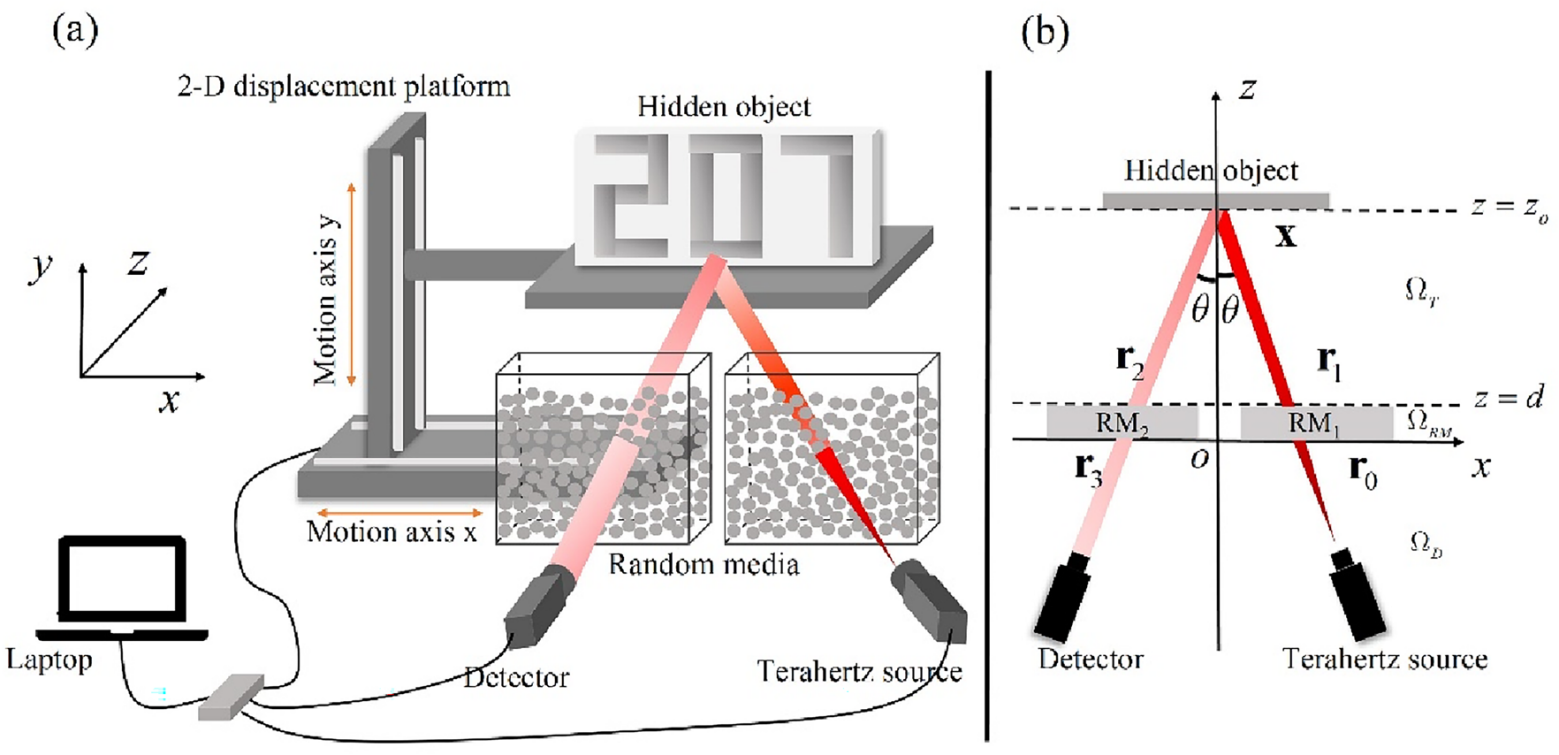
(**a**) Experimental setup. The terahertz waves are emitted from a terahertz source, scattered by the random media, and propagate in free space towards the hidden object. They reflect off the hidden object, pass through random media again, and finally reach the detector's receiving aperture for detection. The 2-D displacement platform moves the hidden object within a 32 $\times$ 32 grid area (approximately 10 cm $\times$ 5 cm), allowing the terahertz waves to illuminate different locations on the hidden object while the detector collects the corresponding time-domain waveforms. The random media is composed of an acrylic container filled with polypropylene plastic particles, with lengths along *x*, *y*, *z* axes of 10 cm, 10 cm, and 3 cm, respectively. A laptop is used to control the 2-D displacement platform and the transmission and reception of terahertz waves (**b**) Top view of the experimental setup. Set the *z* = 0 plane as the surface of the random media near the detector side. The terahertz source and detector are fixed in the detection region $\Omega_{D}$ and symmetric about the *x* = 0 plane. The center lines of the terahertz beam and detector’s detection region are aimed at (0, 0,$z_{o}$), forming an acute angle of $\theta$ with the *x* = 0 plane. The random media is placed in the random media region $\Omega_{RM}$ located between the target and terahertz time-domain spectroscopy system. The hidden object is placed in the target region $\Omega_{T}$ with its geometric center located at (0, 0,$z_{o}$).

Our experimental setup, illustrated in Fig. 1, employs a bistatic terahertz time-domain spectroscopy (TDS) system as the terahertz source and detector. In Fig. 1b, we present an overhead view of our experimental setup, depicting the coordinate system utilized in the calculation process, as well as the points, surfaces, and regions of significance. Set the *z* = 0 plane as the surface of the random media near the detector side. The terahertz source and detector are fixed in the detection region $\Omega_{D} = \left\{ {\left( {x,y,z} \right) \in {\mathbb{R}} \times {\mathbb{R}} \times {\mathbb{R}}|z < 0} \right\}$ and symmetrically positioned about the *x* = 0 plane. The center lines of the terahertz beam and detector’s receiving region are pointed towards (0, 0,$z_{o}$), forming an acute angle of $\theta$ with the *x* = 0 plane. In our experiment, we only emit and receive vertically polarized terahertz waves, allowing us to express the electromagnetic field in scalar form in the subsequent text. The random media is situated in the random media region $\Omega_{RM} = \left\{ {\left( {x,y,z} \right) \in {\mathbb{R}} \times {\mathbb{R}} \times {\mathbb{R}}|0 \le z < d} \right\}$ between the target and terahertz time-domain spectroscopy system, while the hidden object is placed in the target region $\Omega_{T} = \left\{ {\left( {x,y,z} \right) \in {\mathbb{R}} \times {\mathbb{R}} \times {\mathbb{R}}|z \ge d} \right\}$ with its geometric center located at (0, 0,$z_{o}$). In our system, $\theta$ is approximately 10°, *z*_*o*_ is 7 cm. By utilizing a 2-D displacement platform, we moved the hidden object across a two-dimensional plane, enabling the terahertz waves to illuminate different locations on the hidden object and allowing the detector to collect the corresponding time-domain waveforms. This process resulted in a set of three-dimensional data regarding the dimensions of *x*, *y*, *t*.

The terahertz TDS system utilized in our experiment is a T-ray 5000 produced by Advanced Photonix. The terahertz source employed in the experiment emitted pulses with a central frequency of 0.3 THz and a full width at half maximum (FWHM) of around 4 ps, featuring ultra-short Gaussian pulses. The detector used had a receiving aperture of 4 cm in diameter. The detector we utilized is the receiver module of the T-ray 5000. This module features a 700 ps temporal gate, a maximum sampling frequency of 100 Hz, and an adjustable optical delay system. To facilitate movement of the hidden object, we employed a 2-D displacement platform that moves hidden object within an M $\times$ N grid area, in our experiment, M and N were both set to 32, resulting in a scanning area of approximately 10 cm $\times$ 5 cm. The hidden objects were cut into special shapes from tin foil and attached to an acrylic board for fixation. Polypropylene plastic particles with an average diameter of 4 mm were used to fill an acrylic container, which measured 10 cm $\times$ 10 cm $\times$ 3 cm along *x*, *y*, *z* axes, serving as the random media. random media RM_1_ is placed between hidden objects and terahertz source, while random media RM_2_ is placed between hidden objects and detector. Both RM_1_ and RM_2_ shared the same composition and configuration in our experiments.

Our imaging scheme operates under the assumption that the signal returned from the hidden object can be temporally separated from the signal directly returned from the random media. When actively probing an object hidden behand random media, a portion of the terahertz waves will directly return from the random media due to the scattering effect, while others will return after reaching the object. In order to avoid interference from signals that directly return from random media and to maximize the reception of signals during a single detection process, we selected the maximum duration that the detection system can receive, specifically 700 ps, as the length of the temporal gate. Additionally, we adjusted the optical delay length of the system to set an appropriate opening time for the time gate in order to block signals that return from random media. When the object is closely adjacent to the random media or submerged within it, the signal returning from random media overlaps with the signal returning from the object, which distorts the latter signal. Consequently, it is essential to carefully adjust the opening time of the temporal gate. In order to mitigate this distortion, one possible solution could be pre-measuring a set of data without the object can be used as background, enabling the elimination of this background signal from the data obtained with the object. To avoid this distortion, the minimum time interval between the signal returning from random media and the signal returning from the object should exceed the full width at half maximum (FWHM) of the signal returning from the object, indicating the object should be separated from the random media by a certain distance, which corresponds to the FWHM of the signal returning from the object times the speed of light. In our experiment, it is necessary for the object to be separated from the random media by a minimum distance of 2 cm. Besides, as the opening time of the time gate and the emission time of terahertz waves are not consistent, we only focus on the relative position of the hidden objects within the time gate, rather than their absolute time position. We set time *t* = 0 as the opening time of the time gate.

### Imaging method

The complete propagation path of terahertz wave is illustrated in Fig. 1b. To begin with, the terahertz wave emits from the terahertz source, reaches a point ${\mathbf{r}}_{0} \in {\mathbf{S}}_{0} = \left\{ {\left( {r_{0,x} ,r_{0,y} ,r_{0,z} } \right) \in {\mathbb{R}} \times {\mathbb{R}} \times {\mathbb{R}}|r_{0,z} = 0} \right\}$ in the *z* = 0 plane. In the bistatic terahertz TDS system we employed, the terahertz source field distribution is in the form of a Gaussian pulse, but terahertz waves are not normally incident random media, instead, the terahertz beam forms an angle $\theta$ with the *z*-axis. Therefore, the terahertz source field distribution needs to be projected onto the coordinate adopted by our system. Here, we derive the *z* = 0 plane electric field distribution $E_{0} \left( {t,{\mathbf{r}}_{0} } \right)$ in the bistatic terahertz TDS system.

To calculate $E_{0} \left( {t,{\mathbf{r}}_{0} } \right)$, the terahertz source field distribution in the *x* direction needs to be multiplied by a factor of $\cos \theta$, and The propagation factor $t - \left( {{\mathbf{r}}_{0,z} - {\mathbf{r}}_{{0,z_{0} }} } \right)/c_{0}$ is replaced by $t - \left( {\frac{{\left( {{\mathbf{r}}_{0,z} - {\mathbf{r}}_{{0,z_{0} }} } \right) - \left( {{\mathbf{r}}_{0,x} - {\mathbf{r}}_{{0,x_{0} }} } \right)\tan \theta }}{{\sqrt {1 + \tan^{2} \theta } }}} \right)/c_{0}$, in which $\left| {\frac{{\left( {{\mathbf{r}}_{0,z} - {\mathbf{r}}_{{0,z_{0} }} } \right) - \left( {{\mathbf{r}}_{0,x} - {\mathbf{r}}_{{0,x_{0} }} } \right)\tan \theta }}{{\sqrt {1 + \tan^{2} \theta } }}} \right|$ is the distance between the point $\left( {{\mathbf{r}}_{0,x} ,{\mathbf{r}}_{0,y} ,{\mathbf{r}}_{0,z} } \right)$ and the equiphase plane $r_{0,z} = \tan \theta \left( {r_{0,x} - r_{{0,x_{0} }} } \right) + r_{{0,z_{0} }}$. $c_{0}$ denotes the speed of light in vacuum. Taking these factors into account, the distribution of $E_{0} \left( {t,{\mathbf{r}}_{0} } \right)$ can be expressed as:

1 $$\begin{aligned} E_{0} (t,{\mathbf{r}}_{0} ) = & \exp \left( { - \frac{{\left( {\left( {{\mathbf{r}}_{0,x} - {\mathbf{r}}_{{0,x_{0} }} } \right)\cos \theta } \right)^{2} + \left( {{\mathbf{r}}_{0,y} - {\mathbf{r}}_{{0,y_{0} }} } \right)^{2} }}{{2\sigma_{xy}^{2} }}} \right) \\ & \times \exp \left( { - \frac{{\left( {t - \left( {\frac{{\left( {{\mathbf{r}}_{0,z} - {\mathbf{r}}_{{0,z_{0} }} } \right) - \left( {{\mathbf{r}}_{0,x} - {\mathbf{r}}_{{0,x_{0} }} } \right)\tan \theta }}{{\sqrt {1 + \tan^{2} \theta } }}} \right)/c_{0} } \right)^{2} }}{{2\sigma_{t}^{2} }}} \right)\cos \left( {\omega_{0} t} \right) \\ \end{aligned}$$

whereas $\sigma_{xy}$ represents the standard deviations of the Gaussian pulse distribution in dimensions *x*, *y*, and $\sigma_{t}$ represents the standard deviations of the Gaussian pulse distribution in dimensions *t*. $\omega_{0}$ denotes the main frequency of the Gaussian pulse.

Subsequently, the terahertz wave travels through RM_1_ and arrives at a point ${\mathbf{r}}_{1} \in {\mathbf{S}}_{d} = \left\{ {\left( {r_{1,x} ,r_{1,y} ,r_{1,z} } \right) \in {\mathbb{R}} \times {\mathbb{R}} \times {\mathbb{R}}|r_{1,z} = d} \right\}$ situated in the *z* = *d* plane of $\Omega_{RM}$. The electric filed distribution $E_{1} \left( {t,{\mathbf{r}}_{0} } \right)$ in this *z* = *d* plane can be expressed as:

2 $$E_{1} \left( {t,{\mathbf{r}}_{1} } \right) = E_{0} (t,{\mathbf{r}}_{0} ) \otimes h_{{{\mathbf{RM}}_{1} }} (t,{\mathbf{r}}_{0} ,{\mathbf{r}}_{1} ){ = }\int_{{{\mathbf{S}}_{0} }} {\int_{0}^{\infty } {h_{{{\mathbf{RM}}_{1} }} \left( {t - t^{\prime},{\mathbf{r}}_{1} - {\mathbf{r}}_{0} } \right)E_{0} \left( {t^{\prime},{\mathbf{r}}_{0} } \right)dt^{\prime}d{\mathbf{r}}_{0} } }$$

while $h_{{{\mathbf{RM}}_{1} }} (t,{\mathbf{r}}_{0} ,{\mathbf{r}}_{1} )$ is the point spread function of the random media RM_1_.

The scattered terahertz wave further propagates towards a point ${\mathbf{x}} \in \Psi = \left\{ {\left( {x,y,z} \right) \in {\mathbb{R}} \times {\mathbb{R}} \times {\mathbb{R}}|z \ge d} \right\}$ located on the hidden object. After reflecting off the hidden object, it reaches a point ${\mathbf{r}}_{2} \in {\mathbf{S}}_{d} = \left\{ {\left( {r_{1,x} ,r_{1,y} ,r_{1,z} } \right) \in {\mathbb{R}} \times {\mathbb{R}} \times {\mathbb{R}}|r_{1,z} = d} \right\}$ situated in the *z* = *d* plane. The electric filed distribution in the *z* = *d* plane can be described as follows:

3 $$E_{2} \left( {t,{\mathbf{r}}_{2} } \right) = \chi_{{\text{o}}} \left( {t,{\mathbf{r}}_{2} - {\mathbf{r}}_{1} } \right) \otimes E_{1} \left( {t,{\mathbf{r}}_{1} } \right){ = }\int_{{{\mathbf{S}}_{d} }} {\int_{0}^{\infty } {\chi_{{\text{o}}} \left( {t - t^{\prime},{\mathbf{r}}_{2} - {\mathbf{r}}_{1} } \right)E_{1} \left( {t^{\prime},{\mathbf{r}}_{1} } \right)dt^{\prime}d{\mathbf{r}}_{1} } }$$

where $\chi_{{\text{o}}} \left( {t,{\mathbf{r}}_{2} - {\mathbf{r}}_{1} } \right)$ is the point spread function of the target region $\Omega_{T}$. We make an approximation that the main lobe distribution of terahertz wave propagates almost unchanged in the target region. In other words, we consider $\chi_{{\text{o}}} \left( {t,{\mathbf{r}}_{2} - {\mathbf{r}}_{1} } \right)$ to be the hidden object reflectance distribution $R\left( {{\mathbf{x}};\theta } \right)$ in our system, in which the center lines of the terahertz beam and detector’s receiving region are aimed at (0, 0,$z_{o}$), and forms an acute angle of $\theta$ with the *x* = 0 plane as shown in Fig. 1b.

The terahertz waves travel through RM_2_ and arrive at a point ${\mathbf{r}}_{3} \in {\mathbf{S}}_{0} = \left\{ {\left( {r_{0,x} ,r_{0,y} ,r_{0,z} } \right) \in {\mathbb{R}} \times {\mathbb{R}} \times {\mathbb{R}}|r_{0,z} = 0} \right\}$ in the surface *z* = 0. Eventually, terahertz waves reach the detector's receiving aperture for detection, as illustrated in the Fig. 1b. Consequently, the electric field received by the detector can be represented as:

4 $$E_{detection} \left( {t,{\mathbf{r}}_{3} } \right) = h_{{{\mathbf{RM}}_{2} }} (t,{\mathbf{r}}_{2} ,{\mathbf{r}}_{3} ) \otimes E_{2} \left( {t,{\mathbf{r}}_{2} } \right){ = }\int_{{{\mathbf{S}}_{d} }} {\int_{0}^{\infty } {h_{{{\mathbf{RM}}_{2} }} \left( {t - t^{\prime},{\mathbf{r}}_{3} - {\mathbf{r}}_{2} } \right)E_{2} \left( {t^{\prime},{\mathbf{r}}_{2} } \right)dt^{\prime}d{\mathbf{r}}_{2} } }$$

where $h_{{{\mathbf{RM}}_{2} }} (t,{\mathbf{r}}_{2} ,{\mathbf{r}}_{3} )$ is the point spread functions of the random media RM_2_. The simplified convolution form of $E_{detection} \left( {t,{\mathbf{r}}_{3} } \right)$ can be expressed as:

5 $$E_{detection} \left( {t,{\mathbf{r}}_{3} } \right) = E_{0} (t,{\mathbf{r}}_{0} ) \otimes h_{{{\mathbf{RM}}_{1} }} (t,{\mathbf{r}}_{0} ,{\mathbf{r}}_{1} ) \otimes R\left( {{\mathbf{x}};\theta } \right) \otimes h_{{{\mathbf{RM}}_{2} }} (t,{\mathbf{r}}_{2} ,{\mathbf{r}}_{3} )$$

$\otimes$ represents a three-dimensional convolution operation along *x*, *y*, *t* dimensions. Based on Eq. (5), it is easy to use a Wiener deconvolution procedure to recover the hidden object reflectance distribution $R\left( {{\mathbf{x}};\theta } \right)$. Let $T(t,{\mathbf{r}}_{0} ,{\mathbf{r}}_{1} ,{\mathbf{x}},{\mathbf{r}}_{2} ,{\mathbf{r}}_{3} )$ represents $E_{0} (t,{\mathbf{r}}_{0} ) \otimes h_{{{\mathbf{RM}}_{1} }} (t,{\mathbf{r}}_{0} ,{\mathbf{r}}_{1} ) \otimes h_{{{\mathbf{RM}}_{2} }} (t,{\mathbf{r}}_{2} ,{\mathbf{r}}_{3} )$. e is given by:

$$\hat{R}\left( {{\mathbf{x}};\theta } \right) = \mathcal{F}^{ - 1} \left\{ {\mathcal{F}\left\{ {E_{detection} \left( {t,{\mathbf{r}}_{3} } \right)} \right\} \cdot \frac{{\mathcal{F}\left\{ {T(t,{\mathbf{r}}_{0} ,{\mathbf{r}}_{1} ,{\mathbf{x}},{\mathbf{r}}_{2} ,{\mathbf{r}}_{3} )} \right\}^{*} }}{{\left| {\mathcal{F}\left\{ {T(t,{\mathbf{r}}_{0} ,{\mathbf{r}}_{1} ,{\mathbf{x}},{\mathbf{r}}_{2} ,{\mathbf{r}}_{3} )} \right\}} \right|^{2} + \frac{1}{snr}}}} \right\}$$

where $\mathcal{F}$ and $\mathcal{F}^{ - 1}$ represents the Fourier transform and inverse Fourier transform, while the superscript “$*$” denotes the complex conjugate operation. The operator $\left| \cdot \right|^{2}$ performs the calculation of the squared magnitude.$\left| {\mathcal{F}\left\{ T \right\}} \right|^{2} = \mathcal{F}\left\{ T \right\} \cdot \mathcal{F}\left\{ T \right\}^{*}$. Additionally, *snr* refers to the ratio of the signal power spectrum to the noise power spectrum. However, it is often challenging to obtain prior knowledge about these spectrums during the imaging process through random media. In such cases, a commonly used approximation method is to set *snr* as a constant value. 

## Results and discussion

We propose an explicit random media point spread function model $h_{{{\mathbf{RM}}_{1} }} (t,{\mathbf{r}}_{0} ,{\mathbf{r}}_{1} )$ based on the work of Guy Satat et al.^35^. In our experiment, both random media RM_1_ and RM_2_ share the same size. Therefore, we assume that the form of $h_{{{\mathbf{RM}}_{2} }} (t,{\mathbf{r}}_{2} ,{\mathbf{r}}_{3} )$ is consistent with $h_{{{\mathbf{RM}}_{1} }} (t,{\mathbf{r}}_{0} ,{\mathbf{r}}_{1} )$.

6 $$h_{{{\mathbf{RM}}_{1} }} (t,{\mathbf{r}}_{0} ,{\mathbf{r}}_{1} ) = \alpha \cdot f_{T} (t) \cdot W({\mathbf{r}}_{0} ,{\mathbf{r}}_{1} |t) \cdot F\left( {\Delta \omega } \right)$$

here, $\alpha$ is a field intensity scaling factor, $f_{T} (t)$ denotes the probability density distribution function of the detected terahertz field at time *t*, $f_{T} (t)$ describes the broadening of the temporal distribution of terahertz pulses when propagating through random media. $W(x,y|t)$ represents the probability density distribution function of the detected terahertz field at position (*x*, *y*) given the time *t*, $W(x,y|t)$ describes the broadening of the spatial distribution (*x*, *y*) of terahertz pulses when propagating through random media. As a result of the greater scattering and absorption of high-frequency terahertz waves in random media compared to low-frequency terahertz waves. This effect significantly suppresses the high-frequency components of the terahertz waves while allowing the low-frequency components to pass through. In Eq. (6), $F\left( {\Delta \omega } \right)$ is low-pass filter for terahertz waves. We assume that the detected terahertz wave spectrum has the same Gaussian shape as the incident terahertz wave, By fitting the measurements, we obtained the center frequency of measurements as $\omega_{d} = \omega_{0} - \Delta \omega$.$f_{T} (t)$ and $W({\mathbf{r}}_{0} ,{\mathbf{r}}_{1} |t)$ are both normalized. $W({\mathbf{r}}_{0} ,{\mathbf{r}}_{1} |t)$ refers to a time-correlated Gaussian diffusion kernel, which can be expressed in the following form:

7 $$W({\mathbf{r}}_{0} ,{\mathbf{r}}_{1} |t) = \exp \left( { - \frac{{\left\| {{\mathbf{r}}_{1} - {\mathbf{r}}_{0} } \right\|^{2} }}{{4Dc(t - t_{0} )}}} \right)$$

where *D* is a parameter related to the frequency of terahertz wave, as well as the scattering coefficient and absorption coefficient of the random media, *c* denotes the speed of light inside the random media,$c = {{c_{0} } \mathord{\left/ {\vphantom {{c_{0} } n}} \right. \kern-0pt} n}$, while *n* represents the refractive index of random media, $t_{0}$ accounts for the offset of the hidden object signal relative to the time gate opening time, and its specific value is determined through actual measurement data. Similarly, we follow the same approach as Guy Satat et al.^35^ to determining the specific value of parameter *D* using a line search method.

The distribution form of $f_{T} (t)$ is given by:

8 $$f_{T} (t) = \exp \left( { - \frac{{\left( {t - t_{0} } \right)^{2} }}{{2\sigma ^{{_{t}^{{\prime 2}} }} }}} \right)$$

$\sigma_{t}^{\prime }$ is the standard deviation of $f_{T} (t)$.

The parameters used in the experiment for the above-mentioned model are as follows:$\alpha$ is $9 \times 10^{ - 5}$ V/m, *D* is $2.2 \times 10^{ - 4}$ m, $c_{0}$ is $3 \times 10^{8}$ m/s, $\omega_{0}$ is $2.5 \times 10^{11}$ Hz, $\Delta \omega$ is $1.7 \times 10^{11}$ Hz, $\sigma^{\prime}_{t}$ is $5.1 \times 10^{ - 12}$ s. $\sigma_{t}$ is $1.7 \times 10^{ - 12}$ s, $\sigma_{xy}$ is $2.1 \times 10^{ - 3}$ m.

**Figure 2:**
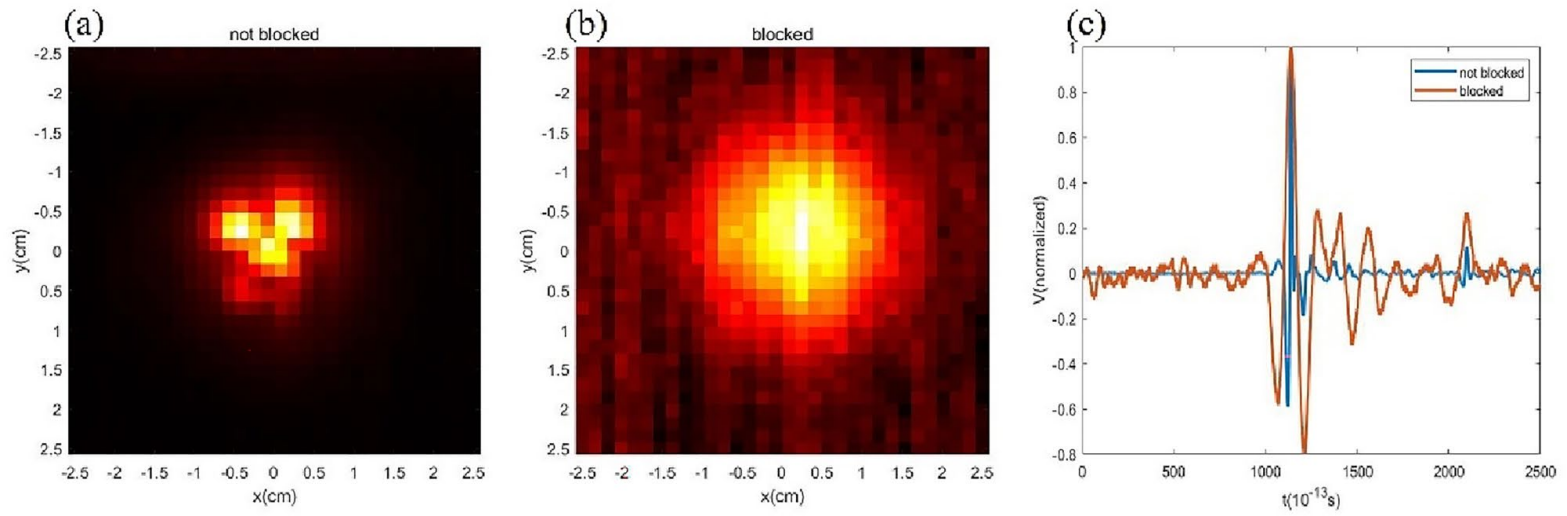
(**a**) The distribution of the detection field when the target is not blocked by RM1 and RM2. (**b**) The detection field distribution when the target is blocked by RM1 and RM2. (**c**) The comparison of the time-domain distribution of the detection field before and after the target is blocked. For ease of presentation, we take the maximum projection in the *t* direction in (**a**,**b**).

To test the distortion effect of polypropylene plastic particles on terahertz waves, we first used a square aluminum foil with a side length of 1.5cm as the target, and measured the detection field distribution in the presence of RM_1_ and RM_2_ simultaneously, as shown in the Fig. 2. Figure 2a shows the distribution of the detection field when the target is not blocked by RM_1_ and RM_2_. Due to the wrinkles in the aluminum foil, the detection field distribution image shows an irregular shape. Figure 2b shows the detection field distribution when the target is blocked by RM_1_ and RM_2_. Figure 2c shows the comparison of the time-domain distribution of the detection field before and after the target is blocked. For ease of presentation, we take the maximum projection in the *t* direction in Fig. 2a,b. The full width at half maximum of Fig. 2b is 1.5cm which is the side length of the square aluminum foil. In Fig. 2c, the full width at half maximum of the detection field distribution when the target is blocked by RM_1_ and RM_2_ is three times greater than the target is not blocked. Due to the scattering effect of the random media, the maximum intensity value of Fig. 2b is less than 2% of that in Fig. 2a. Additionally, we have computed the Structural Similarity Index measure (SSIM) between Fig. 2a and b. SSIM evaluates the similarity between two images by computing brightness, contrast, and structural metrics. The range of SSIM values is from 0 to 1, where higher values indicate greater similarity. The SSIM index between Fig. 2a and b is only 0.1434, indicating a significantly low level of similarity.

**Figure 3:**
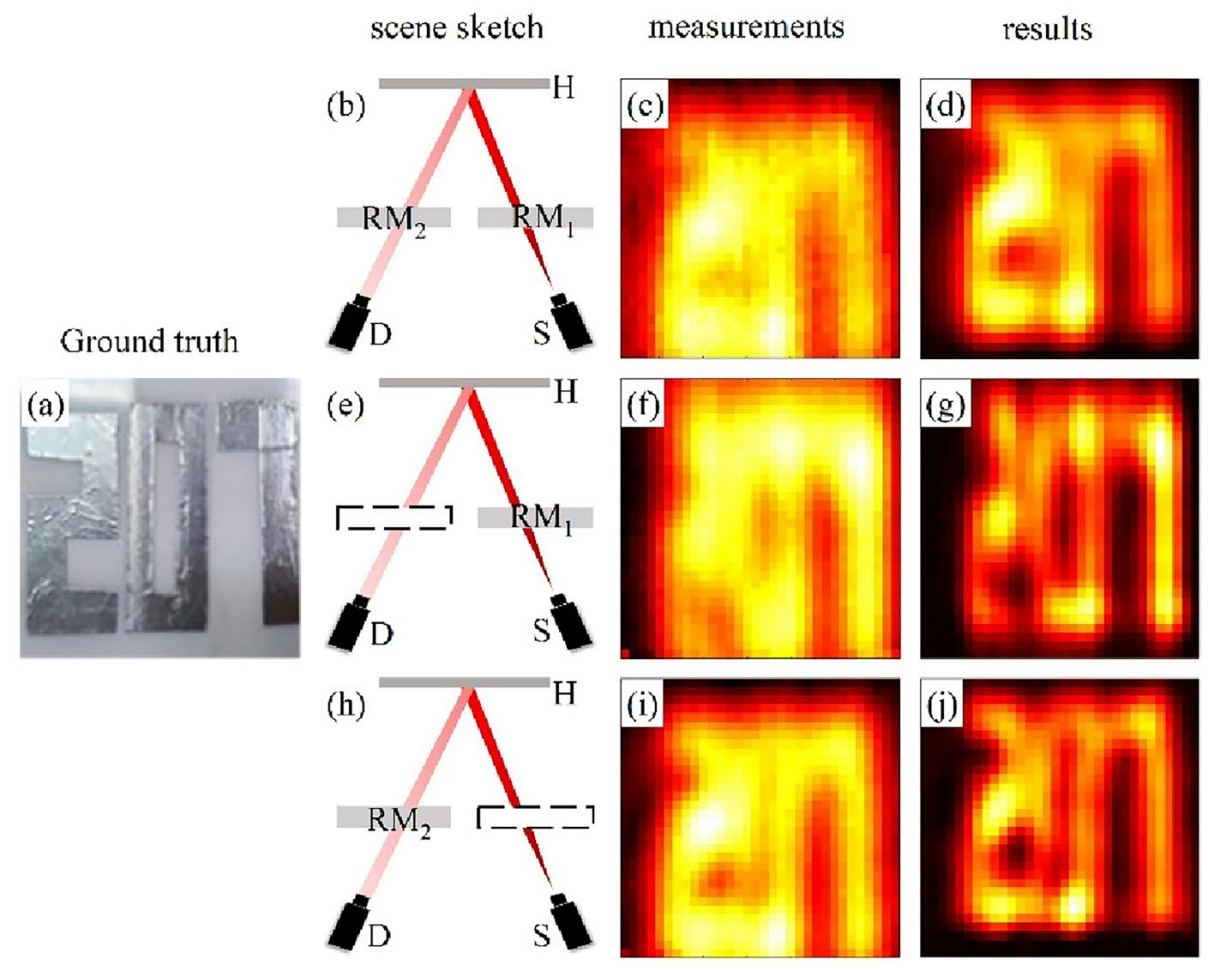
The recovery results of the numbers “207” in the three scenarios. (**a**) The ground truth of 207. (**b**–**d**) The schematic diagram of Scene1, the detected field distribution of numbers “207” in Scene1, and the recovered hidden object reflectance distribution, respectively. (**e**–**g**) The schematic diagram of Scene2, the detected field distribution of numbers “207” in Scene2, and the recovered hidden object reflectance distribution, respectively. (**h**–**j**) Schematic diagram of Scene3, the detected field distribution of numbers “207” in Scene3, and the recovered hidden object reflectance distribution, respectively. In the experiment, all detection data and computations are performed in three dimensions (*x*, *y*, *t*). For ease of presentation, we take the maximum projection in the *t* direction of the three-dimensional matrix. *H* Hidden object, *D* detector, *S* terahertz source.

We examine our imaging scheme in three imaging scenarios, as follows: Scene1, where both the terahertz source and detector are completely blocked by random media; Scene2, where only the terahertz source is blocked by random media; and Scene3, where only the detector is blocked by random media. These scenarios correspond to the presence of random media RM_1_, the presence of random media RM_2_, and the presence of both RM_1_ and RM_2_. When the random media is absent, we assume that the field distribution remains almost unchanged as terahertz waves propagate through this area. In Fig. 3, we display the recovered results of the numbers “207” in all three scenarios. These numbers are cut from tin foil, pasted on an acrylic board, and placed parallel to the *x-o-y* plane. In the experiment, all detection data and computations are carried out in three dimensions (*x*, *y*, *t*). For convenience in presentation, we take the maximum projection in the *t* direction of the three-dimensional matrix.

The results demonstrate that our proposed TBTCI method effectively recovers numbers “207” in the three scenarios, and improves the resolution of the numbers “207”, particularly in distinguishing numbers 2 and 0 in Scene2 and Scene3. Figure 3g,j illustrate that our method exhibits similar recovery ability in Scene2 and Scene3, which validates the rationality of using the same point spread function at RM_2_ and RM_1_. In Scene 1, due to the simultaneous presence of RM_1_ and RM_2_, the terahertz waves pass through the random media twice, resulting in a significantly weakened detection signal, reduced signal-to-noise ratio, and slight poor recovery result. Nevertheless, our method still enables the numbers “2” and “7” to be discernible.

**Figure 4:**
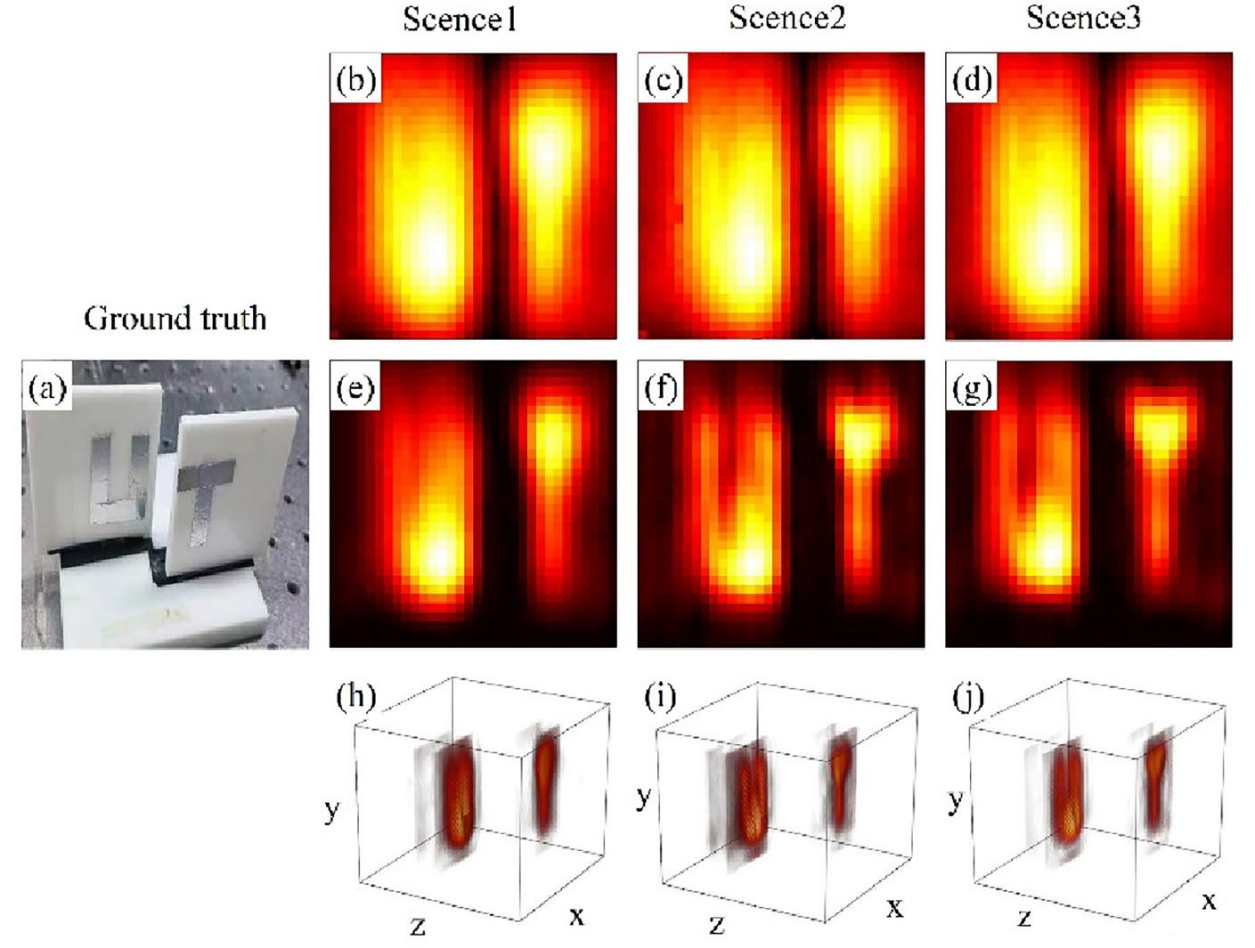
(**a**) The ground truth of the letters “UT” located on different *z* planes with a separation distance of 2 cm, (**b**–**d**) The detected field distribution of the letters “UT” in the three scenarios, (**e**–**g**) The recovered hidden object reflectance distribution of the letters “UT” in the three scenarios, and (**h**–**j**) A three-dimensional visualization of the recovery results for the letters “UT”.

**Figure 5:**
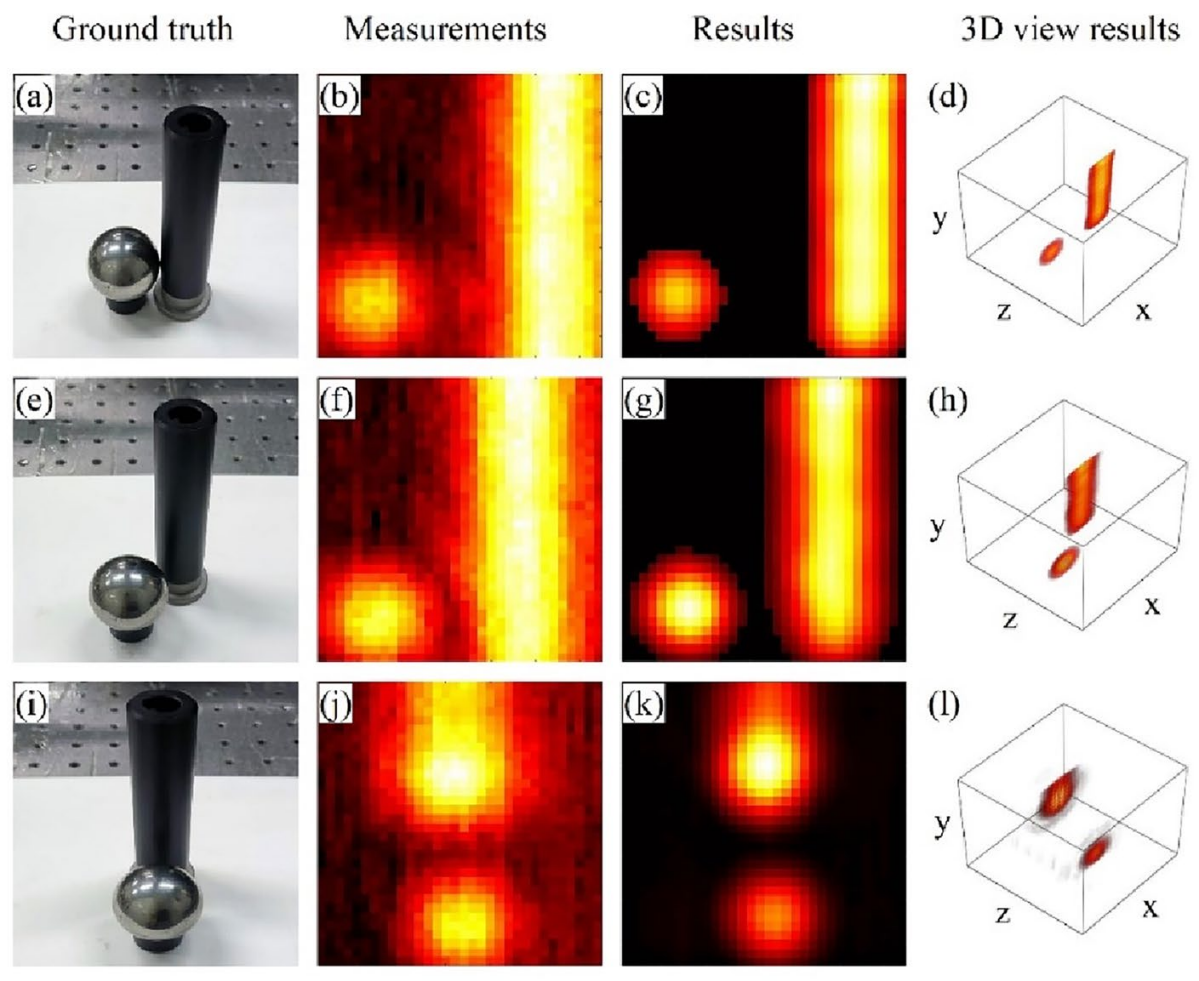
(**a**–**d**) The ground truth, the detected field distribution, the recovered reflectance distribution, and a three-dimensional visualization of the results when the sphere and cylinder are placed side by side with their front surfaces in the same *z*-plane. (**e**–**h**) The ground truth, the detected field distribution, the recovered reflectance distribution, and a three-dimensional visualization of the results when the sphere is placed on the side of cylinder and is closer to the detector. (**i**–**l**) The ground truth, the detected field distribution, the recovered reflectance distribution, and a three-dimensional visualization of the results when the sphere is in front of the cylinder.

Moreover, we also examined the recovery capability of our method for volumetric objects. These volumetric objects include the letters “UT”, which were created by cutting tin foil and affixing them to acrylic boards. They were then positioned on different *z* planes with a separation distance of 2 cm. Additionally, a combination of a metal cylinder and a metal sphere. The corresponding recovery results are shown in Figs. 4 and 5.

Figure 4 demonstrates that our method performs well in recovering objects situated on various planes. The concave portion in the middle of the letter “U” and the edges of the letter “T” exhibit sharpness and clarity in the recovery results. Furthermore, From the recovery results, we determine that the distance between two letters is 2.02 cm, however, due to the utilization of a bistatic measurement method, this distance needs to be divided by a factor of $\cos \theta$. Therefore, the recovered distance $\Delta \hat{z}$ is 2.05 cm. 

In the case of the combination of a metal cylinder and a metal sphere, we conducted imaging experiments of these objects in Scene1 by adjusting their relative positions, as shown in Fig. 5. These relative positions are as follows: the sphere and cylinder are placed side by side with their front surfaces in the same *z*-plane; the sphere is placed on the side of cylinder, closer to the detector; and the sphere is in front of the cylinder. Due to the smooth surfaces of these objects, they predominantly reflect terahertz waves through specular reflection. Consequently, only the surfaces approximately perpendicular to the *z*-axis can be accurately reconstructed. As the metal sphere's surface normal changes along the *x* and *y* axes, it appears as a bright spot in the recovery results. On the other hand, the metal cylinder does not exhibit a surface normal change along the *y*-axis, resulting in a bright band representation. When the cylinder is obstructed by the sphere, there will be some discontinuous reflectivity areas in the recovery results, such as those observed in Fig. 5,k. Although these areas are adjacent in the ground truth image, the non-specular reflection portion of the metal sphere reflects terahertz waves in directions that cannot be detected. Our method effectively suppresses noise, enabling the recovery of reflectance variations with respect to the metal surface's normal direction, as well as the spatial positions of the reflection surfaces.

## Conclusion

In this study, we conducted imaging experiments on objects obscured by random media using a bistatic terahertz time-domain spectroscopy system. By introducing an explicit point spread function of random media, we recover the image of hidden object obscured by the random media. We investigated three types of hidden objects under three random media occlusion scenarios, the experimental results convincingly demonstrate the effectiveness of our imaging method in recovering the images of these hidden objects. Our proposed technique, known as terahertz bistatic three-dimensional computational imaging (TBTCI) through random media offers a solution for imaging in the millimeter-wave range, particularly for applications such as non-invasive testing and biological imaging.

## Data Availability

The datasets generated during and/or analyzed during the current study are available from the corresponding author on reasonable request.

## References

[1] Ishimaru, A. Wave propagation and scattering in random media and rough surfaces. *Proc. IEEE* **79**, 1359–1366. 10.1109/5.104210 (1991).

[2] Goodman, J. W., Huntley, W. H., Jackson, D. W. & Lehmann, M. Wavefront-reconstruction imaging through random media. *Appl. Phys. Lett.* **8**, 311–313 (1966).

[3] Satat, G., Tancik, M. & Raskar, R. Towards photography through realistic fog. In *2018 IEEE International Conference on Computational Photography (ICCP)*, 1–10 (2018).

[4] Khani, M. E. & Arbab, M. H. Two wavelet-based algorithms for chemical recognition using transmission terahertz spectral imaging through turbid media. In *2020 45th International Conference on Infrared, Millimeter, and Terahertz Waves (IRMMW-THz)*, 1–2 (2020).

[5] Luukanen, A., Appleby, R., Kemp, M. & Salmon, N. In *Terahertz Spectroscopy and Imaging* (eds Kai-Erik, P. *et al.*) 491–520 (Springer, 2013).

[6] Sheen, D. M., McMakin, D. L. & Hall, T. E. Three-dimensional millimeter-wave imaging for concealed weapon detection. *IEEE Trans. Microw. Theory Tech.* **49**, 1581–1592. 10.1109/22.942570 (2001).

[7] Wei, Y. *et al.* Occluded prohibited items detection: An X-ray security inspection benchmark and de-occlusion attention module. In *Proceedings of the 28th ACM International Conference on Multimedia* (2020).

[8] Maccarone, A., Mattioli Della Rocca, F., McCarthy, A., Henderson, R. & Buller, G. S. Three-dimensional imaging of stationary and moving targets in turbid underwater environments using a single-photon detector array. *Opt. Express* **27**, 28437–28456. 10.1364/oe.27.028437 (2019). 31684596 10.1364/OE.27.028437

[9] Zhao, F. *et al.* Metalens-assisted system for underwater imaging. *Laser Photon. Rev.* **15**, 25 (2021).

[10] Yang, X. *et al.* Underwater ghost imaging based on generative adversarial networks with high imaging quality. *Opt. Express* **29**(18), 28388–28405 (2021). 34614971 10.1364/OE.435276

[11] Eggebrecht, A. T. *et al.* Mapping distributed brain function and networks with diffuse optical tomography. *Nat. Photon.* **8**, 448–454. 10.1038/nphoton.2014.107 (2014). 10.1038/nphoton.2014.107 PMC4114252 25083161

[12] Ntziachristos, V. Going deeper than microscopy: The optical imaging frontier in biology. *Nat. Methods* **7**, 603–614. 10.1038/nmeth.1483 (2010). 20676081 10.1038/nmeth.1483

[13] Shikhaliev, P. M. Soft tissue imaging with photon counting spectroscopic CT. *Phys. Med. Biol.* **60**, 2453–2474 (2015). 25739788 10.1088/0031-9155/60/6/2453

[14] Li, Q., Zeng, J., Miao, Q. & Gao, M. Self-illuminating agents for deep-tissue optical imaging. *Front. Bioeng. Biotechnol.* **7**, 25 (2019). 31799247 10.3389/fbioe.2019.00326 PMC6861855

[15] Arbab, M. H., Khani, M. E., Harris, Z. B., Virk, A. & Osman, O. B. Terahertz imaging and spectroscopic measurements in the presence of scattering for biophotonics applications. In *2022 47th International Conference on Infrared, Millimeter and Terahertz Waves (IRMMW-THz)*, 1–3 (2022).

[16] Ruan, H., Liu, Y., Xu, J., Huang, Y. & Yang, C. Fluorescence imaging through dynamic scattering media with speckle-encoded ultrasound-modulated light correlation. *Nat. Photon.* **14**, 511–516 (2020).

[17] Indebetouw, G. & Klysubun, P. Imaging through scattering media with depth resolution by use of low-coherence gating in spatiotemporal digital holography. *Opt. Lett.* **25**, 212–214. 10.1364/ol.25.000212 (2000). 18059832 10.1364/ol.25.000212

[18] Wang, L., Ho, P. P., Liu, C., Zhang, G. & Alfano, R. R. Ballistic 2-D imaging through scattering walls using an ultrafast optical kerr gate. *Science* **253**, 769–771. 10.1126/science.253.5021.769 (1991). 17835493 10.1126/science.253.5021.769

[19] Redo-Sanchez, A. *et al.* Terahertz time-gated spectral imaging for content extraction through layered structures. *Nat. Commun.* **7**, 12665. 10.1038/ncomms12665 (2016). 27610926 10.1038/ncomms12665 PMC5023963

[20] Dunsby, C. & French, P. M. W. Techniques for depth-resolved imaging through turbid media including coherence-gated imaging. *J. Phys. D Appl. Phys.* **36**, R207. 10.1088/0022-3727/36/14/201 (2003).

[21] Salhov, O., Weinberg, G. & Katz, O. Depth-resolved speckle-correlations imaging through scattering layers via coherence gating. *Opt. Lett.* **43**, 5528–5531. 10.1364/ol.43.005528 (2018). 30439887 10.1364/OL.43.005528

[22] Shi, T. *et al.* Computational imaging of moving objects obscured by a random corridor via speckle correlations. *Nat. Commun.*10.1038/s41467-022-31669-7 (2022). 35835739 10.1038/s41467-022-31669-7 PMC9283427

[23] Divitt, S., Gardner, D. F. & Watnik, A. T. Imaging around corners in the mid-infrared using speckle correlations. *Opt. Express* **28**, 11051–11064. 10.1364/oe.388260 (2020). 32403624 10.1364/OE.388260

[24] Katz, O., Heidmann, P., Fink, M. & Gigan, S. Non-invasive single-shot imaging through scattering layers and around corners via speckle correlations. *Nat. Photon.* **8**, 784–790. 10.1038/nphoton.2014.189 (2014).

[25] Zhang, W. *et al.* Locating through dynamic scattering media based on speckle correlations. *Appl. Opt.* **61**(35), 10352–10361 (2022). 36607093 10.1364/AO.470271

[26] Lu, T.-F.L.T.-F. *et al.* Non-invasive imaging through dynamic scattering layers via speckle correlations. *Opt. Rev.* **28**, 557–563 (2021).

[27] Li, F., Zhao, M., Tian, Z., Willomitzer, F. & Cossairt, O. Compressive ghost imaging through scattering media with deep learning. *Opt. Express* **28**(12), 17395–17408 (2020). 32679948 10.1364/OE.394639

[28] Zhu, S., Guo, E., Gu, J., Bai, L. & Han, J. Imaging through unknown scattering media based on physics-informed learning. *Photon. Res.* **9**, B210–B219. 10.1364/PRJ.416551 (2021).

[29] Rawat, S., Wendoloski, J. C. & Wang, A. cGAN-assisted imaging through stationary scattering media. *Opt. Express* **30**(11), 18145–18155 (2022). 36221621 10.1364/OE.450321

[30] Sun, Y., Shi, J., Sun, L., Fan, J. & Zeng, G. Image reconstruction through dynamic scattering media based on deep learning. *Opt. Express* **27**, 16032–16046. 10.1364/OE.27.016032 (2019). 31163790 10.1364/OE.27.016032

[31] Sun, L., Shi, J., Wu, X., Sun, Y. & Zeng, G. Photon-limited imaging through scattering medium based on deep learning. *Opt. Express* **27**, 33120–33134. 10.1364/OE.27.033120 (2019). 31878386 10.1364/OE.27.033120

[32] Lai, X., Li, Q., Chen, Z., Shao, X. & Pu, J. Reconstructing images of two adjacent objects passing through scattering medium via deep learning. *Opt. Express* **29**, 43280–43291. 10.1364/OE.446630 (2021).

[33] Lyu, M., Wang, H., Li, G., Zheng, S. & Situ, G. Learning-based lensless imaging through optically thick scattering media. *Adv. Photon.* **1**, 036002–036002 (2019).

[34] Lindell, D. B. & Wetzstein, G. Three-dimensional imaging through scattering media based on confocal diffuse tomography. *Nat. Commun.*10.1038/s41467-020-18346-3 (2020). 32908155 10.1038/s41467-020-18346-3 PMC7481188

[35] Satat, G., Heshmat, B., Raviv, D. & Raskar, R. All photons imaging through volumetric scattering. *Sci. Rep.* **6**, 25. 10.1038/srep33946 (2016). 27683065 10.1038/srep33946 PMC5041145

